# Acupuncture attenuates comorbid anxiety- and depressive-like behaviors of atopic dermatitis through modulating neuroadaptation in the brain reward circuit in mice

**DOI:** 10.1186/s40659-022-00396-0

**Published:** 2022-09-10

**Authors:** Mijung Yeom, Sora Ahn, Sun-Young Jang, Jae-Hwan Jang, Youngrye Lee, Dae-Hyun Hahm, Hi-Joon Park

**Affiliations:** 1 Acupuncture and Meridian Science Research Center (AMSRC), College of Korean Medicine, Kyung Hee University, 02447 Seoul, Republic of Korea; 2 Department of Meridian Medical Science, College of Korean Medicine, Graduate School, Kyung Hee University, 02447 Seoul, Republic of Korea; 3 Department of Physiology, School of Medicine, Kyung Hee University, 02447 Seoul, Republic of Korea; 4 BioNanocomposite Research Center, Kyung Hee University, 02447 Seoul, Republic of Korea; 5 Department of Anatomy & Information Sciences, College of Korean Medicine, Kyung Hee University, 02447 Seoul, Republic of Korea

**Keywords:** Acupuncture, Atopic dermatitis, Itch, Anxiety, Depression, Neuronal adaptation, Striatum

## Abstract

Atopic dermatitis (AD) is highly comorbid with negative emotions such as anxiety and depression. Although acupuncture has demonstrated efficacy in AD, its influence on comorbid anxiety and depression remains unclear. We sought to explore the impact and mechanisms of action of acupuncture on comorbid anxiety and depression of AD. AD-like skin lesions were induced by the topical application of MC903 to the mouse cheek. Acupuncture was performed at Gok-Ji (LI11) acupoints. AD-like phenotypes were quantified by lesion scores, scratching behavior, and histopathological changes. The effects of acupuncture on comorbid anxiety and depression-like behaviors were assessed using the elevated plus-maze (EPM), open-field tests (OFT), and tail-suspension test (TST). In addition, biochemical changes in the brain reward regions were investigated by immunoblotting for the expression of tyrosine hydroxylase (TH), dopamine D1 receptor (D1R), phospho-dopamine and cAMP-regulated phosphoprotein-32 kDa (pDARPP-32), phospho-cAMP response element binding protein (pCREB), ΔFosB, and brain-derived neurotrophic factor (BDNF) in the nucleus accumbens, dorsolateral striatum, and ventral tegmental area. Acupuncture effectively improved the chronic itching and robust AD-like skin lesions with epidermal thickening. Additionally, it considerably reduced comorbid anxiety- and depression-like symptoms, as indicated by more time spent in the open arms of the EPM and in the center of the open field and less time spent immobile in the TST. Higher pCREB, ΔFosB, BDNF, and pDARPP-32 levels, and reduced TH and D1R protein expression in the brain reward regions of AD mice were reversed by acupuncture treatment. The beneficial effects of acupuncture on clinical symptoms (scratching behavior) and comorbid psychological distress in AD strongly correlated with dorsal striatal ΔFosB levels. Collectively, these data indicate that acupuncture had a significant, positive impact on comorbid anxiety- and depression-like behaviors by modulating neuroadaptation in the brain reward circuit in mice with AD, providing a novel perspective for the non-pharmacological management of psychiatric comorbidities of AD.

## Background

Atopic dermatitis (AD) is a common yet complex inflammatory skin disease characterized by intense itching and recurrent eczematous lesions [1] that affects around 15–30% of children and 2–10% of adults, with a highly variable prevalence worldwide [2].

Over the past several years, evidence has shown an association between AD and psychological comorbidities such as anxiety and depression [3, 4], which can lead to reduced health status, health-related quality of life, and work productivity [5, 6]. Although anxiety and depression symptoms are particularly associated with more severe AD [7], even mild AD may be accompanied by these emotional states [8]. Thus, it is increasingly being recognized that anxiety and depression are consistent comorbidities in AD, requiring preventative or early intervention. Indeed, psychotherapy has been reported to be effective in improving mental health concerns and symptoms, such as itching [9]. However, recent studies suggest that these psychological comorbidities may be reduced with improved control of AD signs and symptoms [3, 10–12].

The mechanisms by which AD patients may be predisposed to comorbid psychological conditions remain poorly understood. However, the persistent itch-scratch cycle, the most prominent yet difficult feature to control in patients with AD, has been postulated to be an important contributing factor that leads to increased anxiety and depression in AD. Besides providing itch relief, scratching the itch also evokes a pleasurable experience which can be rewarding and even addictive, especially in chronic itch conditions [13–15]. Subsequently, this induces abnormalities in brain reward circuit, which centers on dopaminergic neurons projecting from the ventral tegmental area (VTA) to the limbic systems, in particular, the dorsal striatum (dStr) and nucleus accumbens (NAcc) [14–18]. Similar to addiction, these abnormalities in the brain reward system have been suggested as a mechanism by which AD elicits negative emotional states [15, 19]. Indeed, we have previously shown that mice with AD display depression- and anxiety-like behaviors with concomitant neuronal adaptations in brain reward circuits [20].

Acupuncture is a promising alternative modality for the treatment of AD amid concerns regarding the potential adverse effects of conventional medications, frustration with the chronic nature of the disease, and dissatisfaction with conventional medical treatment [21]. Multiple systematic reviews have shown that acupuncture is effective in ameliorating itching and clinical signs and symptoms [22–24]. In addition, our previous studies have shown that acupuncture can improve the signs and symptoms of AD in mice and patients with mild-to-moderate AD [25–27]. Thus, it is becoming increasingly popular as a treatment modality for itch and skin conditions and is practiced worldwide [24, 28]. However, it is not known whether acupuncture treatment ameliorates AD-associated anxiety and depression behaviors, and which neurobiological changes are involved in the mechanism.

In the present study, we sought to determine whether acupuncture decreases comorbid anxiety- and depression-like behaviors in MC903-induced AD mice and modulates dopamine- and plasticity-related signaling changes in the mesolimbic reward circuit.

## Results

### Acupuncture attenuated chronic itch as well as atopic dermatitis-like lesions in MP903-induced AD mice

**Fig. 1:**
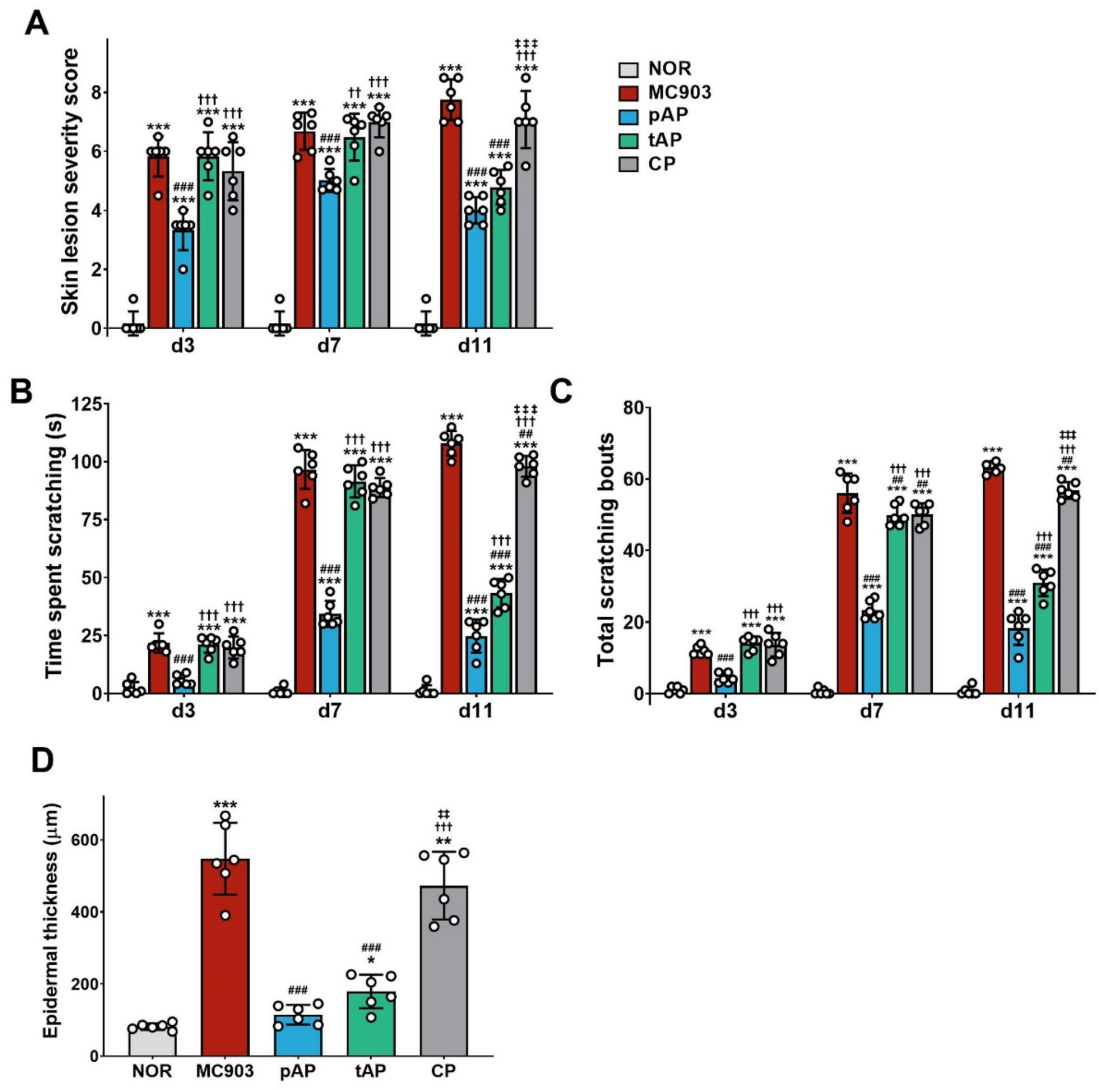
Effects of acupuncture treatment on atopic dermatitis-like skin lesions and chronic persistent itch in MC903-induced atopic dermatitis mice. **A** Time-course of atopic dermatitis-like lesion severity. The severity of skin lesion symptoms (erythema, xerosis and excoriation) was evaluated macroscopically on days 3, 7 and 11. **B**, **C** Time-course of scratching behaviors. Total time spent **B** and number **C** of scratching for 30 min were quantified on days 3, 7 and 11. **D** Measurements of the epidermal thickness from the hematoxylin and eosin–stained sections are shown in Additional file 1: Fig. S1. *NOR* untreated control group, *MC903* MC903-induced atopic dermatitis group, *pAP* MC903- and preventive acupuncture-treated group, *tAP* MC903- and therapeutic acupuncture-treated group, *CP* MC903- and acupuncture at control point (non-acupoint)-treated group. All data are presented as mean ± SD, n = 6 mice/group. ***p < 0.001 vs. NOR group; ^##^*p* < 0.01, ^###^*p* < 0.001 vs. MC903 group; ^††^*p* < 0.01, ^†††^*p* < 0.001 vs. pAT group; ^‡‡^ *p* < 0.01, ^‡‡‡^ *p* < 0.001 vs. tAT group

We first sought to confirm the effectiveness of acupuncture in the treatment of AD-like skin inflammation and itching. Consistent with our previous report [25], we found that both preventive and therapeutic acupuncture treatment significantly attenuated MC903-induced AD severity, whereas acupuncture stimulation at control points had no effect on AD-like skin lesions in mice (Fig. 1A). As is true for AD patients, MC903-treated mice displayed scratching behaviors that began on the third day of MC903 treatment and increased in intensity even after the 7-day treatment period. Preventive acupuncture treatment significantly suppressed the increase in itch intensity over time. Therapeutic acupuncture for four days after the 7-day ongoing treatment period also significantly inhibited the strong scratch response induced by MC903 (Fig. 1B, C). In addition, histological analysis showed that preventive and therapeutic acupuncture treatment markedly inhibited epidermal thickening in mice with MC903-induced AD by 79.0% and 67.2%, respectively. These effects were not observed in mice with stimulation of the control points by acupuncture needles (Fig. 1D and Additional file 1: Figs. S1). Taken together, these results suggest that acupuncture alleviates MC903-induced skin lesions and pruritus.

### Acupuncture decreased concomitant anxiety- and depressive-like behaviors in MC903-induced AD mice

**Fig. 2:**
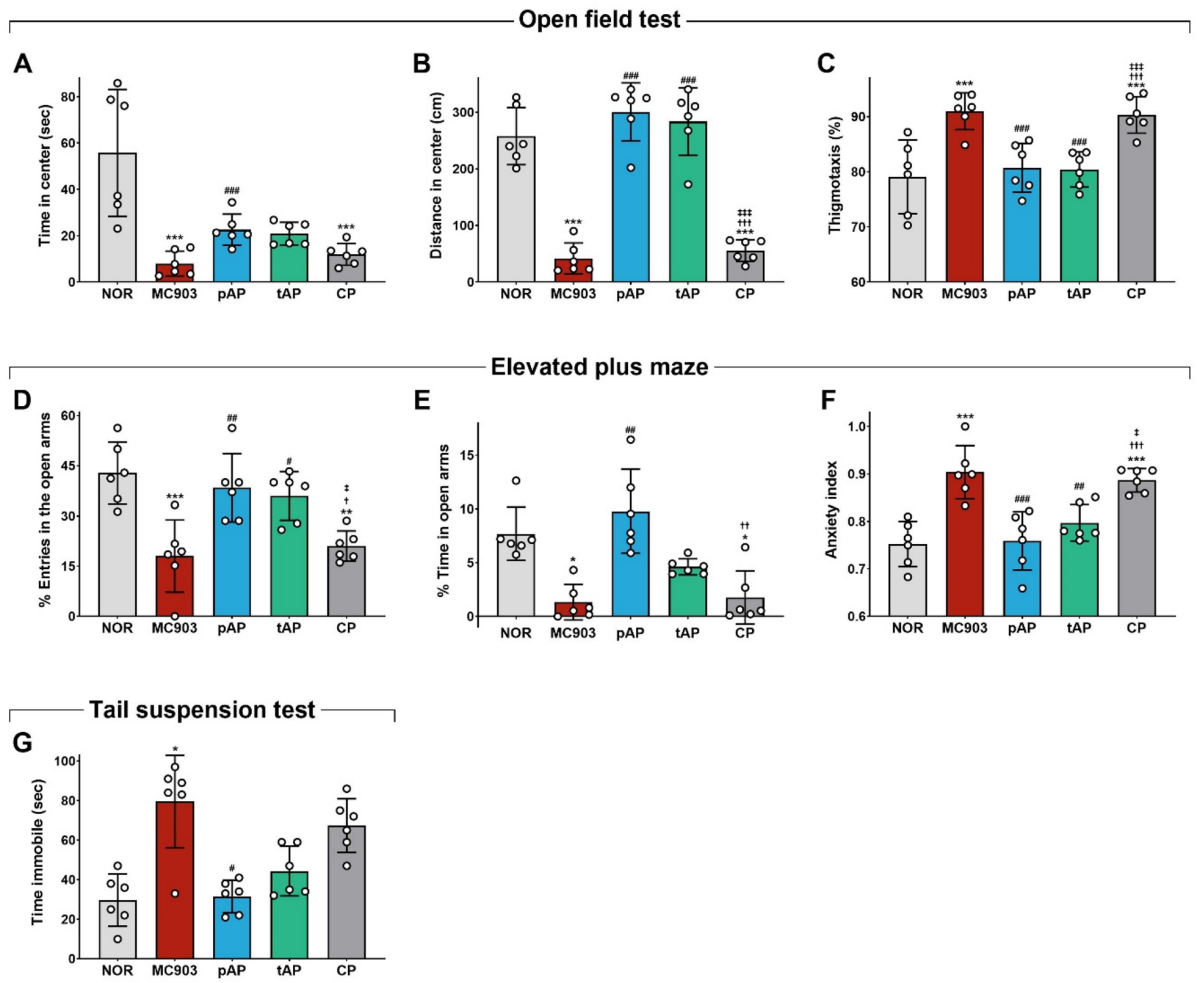
Effects of acupuncture treatment on concomitant anxiety- and depressive-like behaviors in MC903-induced atopic dermatitis mice. Anxiety-like behavior was measured by open field test (OFT; **A**–**C**) and elevated plus maze (EPM; **D**–**F**) on day 10 and depressive-like behavior by the tail suspension test (TST; **G**) on day 11. **A** Time spent in center in OFT. **B** The amount of distance traveled in center in OFT. **C** Thigmotaxis in OFT. **D** Entries (%) into open arms in EPM. **E** Time (%) spent in open arms in EPM. **F** Anxiety index in EPM. **G** Immobility time in the TST. *NOR* untreated control group, *MC903* MC903-induced atopic dermatitis group, *pAP* MC903- and preventive acupuncture-treated group, *tAP* MC903- and therapeutic acupuncture-treated group, *CP* MC903- and acupuncture at control point (non-acupoint)-treated group. All data are presented as mean ± SD, n = 6 mice/group. *p < 0.05, **p < 0.01, ***p < 0.001 vs. NOR group; ^#^*p* < 0.05, ^##^*p* < 0.01, ^###^*p* < 0.001 vs. MC903 group; ^†^*p* < 0.05, ^††^*p* < 0.01, ^†††^*p* < 0.001 vs. pAT group; ^‡^*p* < 0.05, ^‡‡‡^*p* < 0.001 vs. tAT group

We have previously shown that MC903-induced AD elicits anxiety- and depression-like behaviors in mice [20]. Recent studies suggest that effective therapies for skin conditions can ameliorate psychiatric distress, including anxiety and depression [3, 29], with acupuncture becoming widely popular for the treatment of anxiety and depression [30]. Therefore, we next assessed whether acupuncture can reduce concomitant anxiety- and depression-like behaviors in MC903-induced AD mice. To measure anxiety-like behavior, we first used the open field test (OFT) and confirmed our previous observation that AD mice showed little tendency to explore the center of the open field arena, as measured by the time spent (Fig. 2A) and the distance traveled (Fig. 2B) in the central zone. Furthermore, these animals displayed a 15% increase in thigmotaxis (Fig. 2C), which integrated the OFT behavior score (higher scores indicate higher anxiety). Our results demonstrated that both preventive and therapeutic acupuncture treatments significantly reduced AD-induced anxiety-like behaviors. However, stimulation of control points by acupuncture needles did not affect any of the aforementioned parameters in the OFT (Fig. 2A–C). Similarly, in the elevated plus maze (EPM), as previously observed, AD mice avoided entry into the open arms of the EPM compared to normal control mice (Fig. 2D, E), indicating increased anxiety-like behavior. Furthermore, the anxiety index that integrated the EPM behavioral scores was significantly higher in MC903-treated mice than in vehicle-treated controls (Fig. 2F). Consistent with the OFT results, acupuncture treatment significantly decreased the AD-induced anxiety-like phenotype in AD mice, as indicated by entries into (Fig. 2D) and time spent in (Fig. 2E) the open arms of the EPM and the anxiety index (Fig. 2F). However, stimulation of the control points by acupuncture needles did not affect any of the aforementioned parameters in the EPM (Fig. 2D–F).

Next, we assessed depression-like behavior using the tail suspension test (TST), a measure of behavioral despair. MC903-induced AD mice showed a significant increase in behavioral despair when compared to NOR mice, as indicated by the significantly increased time spent immobile in the TST (Fig. 2G). However, mice treated with acupuncture exhibited immobility similar to that of the NOR mice (Fig. 2G). Stimulation of control points by acupuncture needles did not reduce the time spent immobile in the TST (Fig. 2G).

**Fig. 3:**
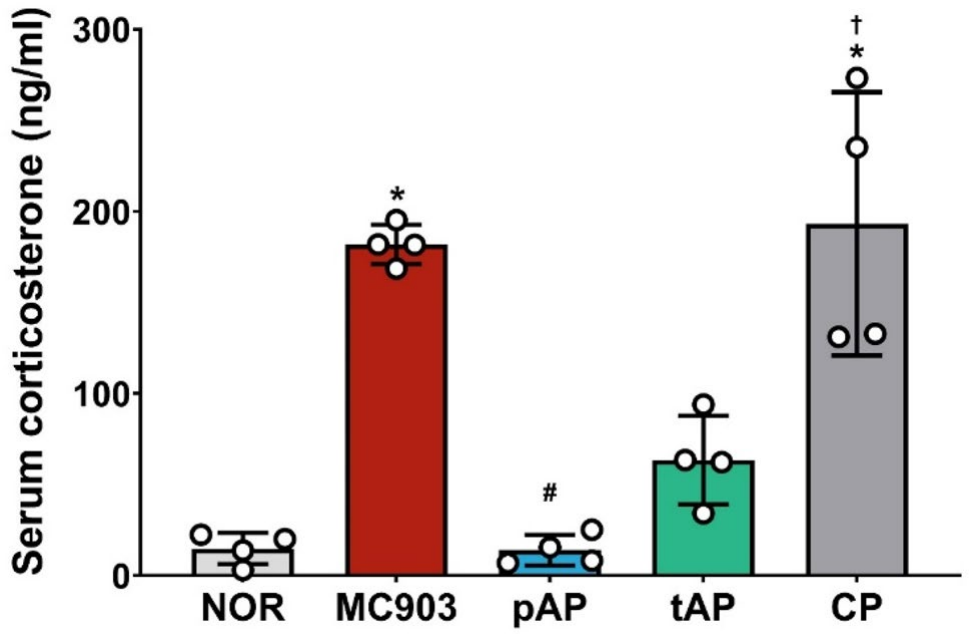
Effects of acupuncture treatment on increased basal serum corticosterone levels in MC903-induced atopic dermatitis mice. Serum corticosterone levels were measured on day 11. *NOR* untreated control group, *MC903* MC903-induced atopic dermatitis group, *pAP* MC903- and preventive acupuncture-treated group, *tAP* MC903- and therapeutic acupuncture-treated group, *CP* MC903- and acupuncture at control point (non-acupoint)-treated group. All data are presented as mean ± SD, n = 4 mice/group. *p < 0.05 vs. NOR group; ^#^*p* < 0.05 vs. MC903 group; ^†^*p* < 0.05 vs. pAT group

To determine whether acupuncture affects basal corticosterone levels in MC903-induced AD mice, we measured serum corticosterone levels. AD mice exhibited elevated basal serum corticosterone levels compared to NOR mice (Fig. 3). Acupuncture treatment significantly reduced basal corticosterone levels in MC903-induced AD mice. In particular, mice administered preventive acupuncture showed a 92.4% reduction in serum corticosterone levels compared with MC903-induced AD mice (Fig. 3). However, acupuncture at the control point showed no positive effects (Fig. 3).

### Acupuncture normalized alterations of plasticity- and dopamine-related signals in the brain reward circuits of MC903-induced AD mice

**Fig. 4:**
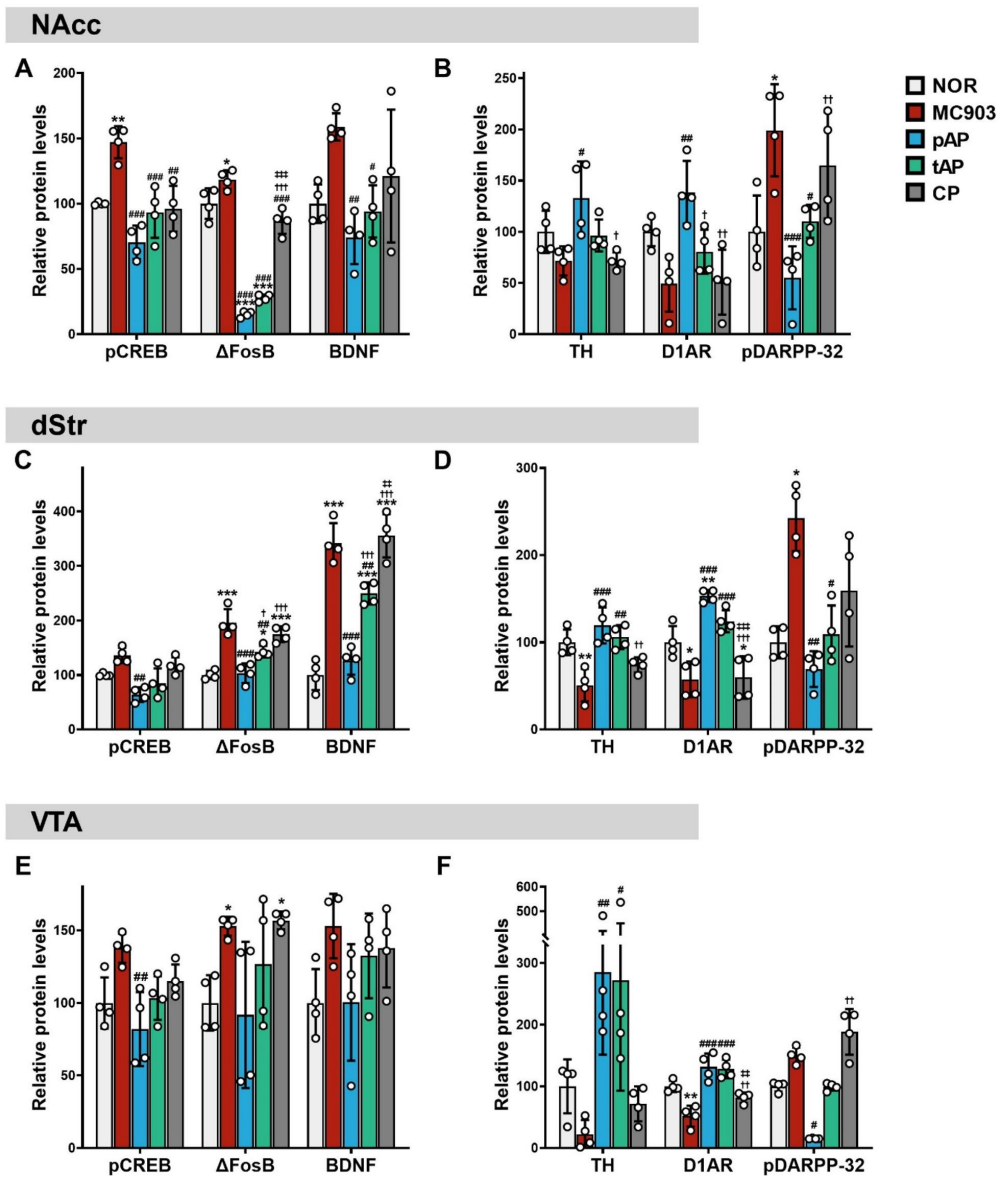
Effects of acupuncture treatment on changes in reward signaling pathway in the brain reward regions of MC903-induced atopic dermatitis mice. Relative expression levels of plasticity-related (**A**, **C**, and **E**; pCREB, ΔFosB and BDNF) and DA-related (**B**, **D**, and **F**; TH, D1AR and pDARPP-32) proteins were determined by immunoblotting analysis of the lysates from the nucleus accumbens (NAcc), dorsal striatum (dStr), and ventral tegmental area (VTA). The abundance of the target protein was normalized to β-actin (for ΔFosB, BDNF, TH and D1AR), total CREB (for pCREB) or total DARPP-32 (for pDARPP-32) and expressed as a percentage change, relative to the NOR. *NOR* untreated control group, *MC903* MC903-induced atopic dermatitis group, *pAP* MC903- and preventive acupuncture-treated group, *tAP* MC903- and therapeutic acupuncture-treated group, *CP* MC903- and acupuncture at control point (non-acupoint)-treated group. *CREB* cyclic AMP-response element binding protein, *pCREB* phospho-Ser133 CREB, *BDNF* brain-derived neurotrophic factor, *TH* tyrosine hydroxylase, *D1AR* dopamine D1 receptor,  *DARPP-32* dopamine- and cAMP-regulated phosphoprotein of 32 kDa, *pDARPP-32* phospho-Thr34 DARPP-32. All data are presented as mean ± SD, n = 4 mice/group. * *p* < 0.05, ** *p* < 0.01, *** *p* < 0.001 vs. NOR group; ^#^*p* < 0.05, ^##^*p* < 0.01, ^###^*p* < 0.001 vs. MC903 group; ^†^*p* < 0.05, ^††^*p* < 0.01, ^†††^*p* < 0.001 vs. pAT group; ^‡‡^*p* < 0.01, ^‡‡‡^*p* < 0.001 vs. tAT group

To determine whether acupuncture blocks alterations in plasticity- and dopamine-related signals in the brain reward circuits of mice with AD and concomitant anxiety- and depression-like symptoms, we measured the expression levels of plasticity- and dopamine-related proteins in the brain reward regions using immunoblotting. In the NAcc of AD mice, there was a significant increase in the phosphorylated levels of cyclic AMP-response element binding protein (CREB), a modest but statistically significant increase in ΔFosB protein levels, and a large but not statistically significant increase in brain-derived neurotrophic factor (BDNF) levels compared to the NOR group (Fig. 4A and Additional file 1: Fig. S3A). We also observed a non-significant trend for lower levels of tyrosine hydroxylase (TH), the rate-limiting enzyme for dopamine biosynthesis, and dopamine D1 receptor (D1AR) protein, and a significant increase in the levels of phospho-Thr34 dopamine- and cAMP-regulated phosphoprotein of 32 kDa (pDARPP-32) protein in the NAcc after MC903 treatment as compared with ethanol vehicle (Fig. 4B and Additional file 1: Fig.S3B). All these changes, except for TH and D1AR levels, were significantly reversed by preventive acupuncture treatment in mice with AD. Therapeutic acupuncture also increased TH and D1AR protein levels, although slightly and not statistically significant, when compared to those of mice with AD. Acupuncture needle stimulation at the control point had no effect (Fig. 4A, B and Additional file 1: Fig. S3A, B). Similar to the results in the NAcc, there was a small and insignificant increase in phospho-Ser133 CREB (pCREB) levels, significant increase in ΔFosB, BDNF and pDARPP-32 protein levels, and substantial decreases in TH and D1AR levels in the dStr. Acupuncture treatment evoked sharp increases in pCREB, ΔFosB, BDNF, and pDARPP-32 and sharp decreases in TH and D1AR levels, whereas acupuncture at non-acupoints had no effect (Fig. 4C, D and Additional file 1: Fig. S3C, D). Finally, similar changes were observed in the VTA. However, mice with MC903-induced AD exhibited significant changes only in ΔFosB and D1AR protein levels compared to NOR mice. When compared to the MC903 group, there were significant decreases in pCREB and pDARPP-32 levels in the pAP group and a significant increase in TH and D1AR levels in the pAP and tAP groups, whereas acupuncture needle stimulation at the control point had no effect (Fig. 4E, F and Additional file 1: Fig. S3E, F).

### Improvement in itch and concomitant anxiety- and depression-like symptoms with acupuncture treatment correlates with changes in reward signaling pathway in the brain reward regions

As expected, the clinical symptoms of AD (skin lesion severity and scratching an itch) were strongly positively correlated with anxiety- and depression-like behavior in the EPM, OFT, and TST (Additional file 1: Fig. S4A). Further, we found that the improvement in skin lesion severity and scratching behavior following acupuncture treatment was accompanied by a reduction in concomitant anxiety- and depression-like symptoms (Additional file 1: Fig. S4B).

**Fig. 5:**
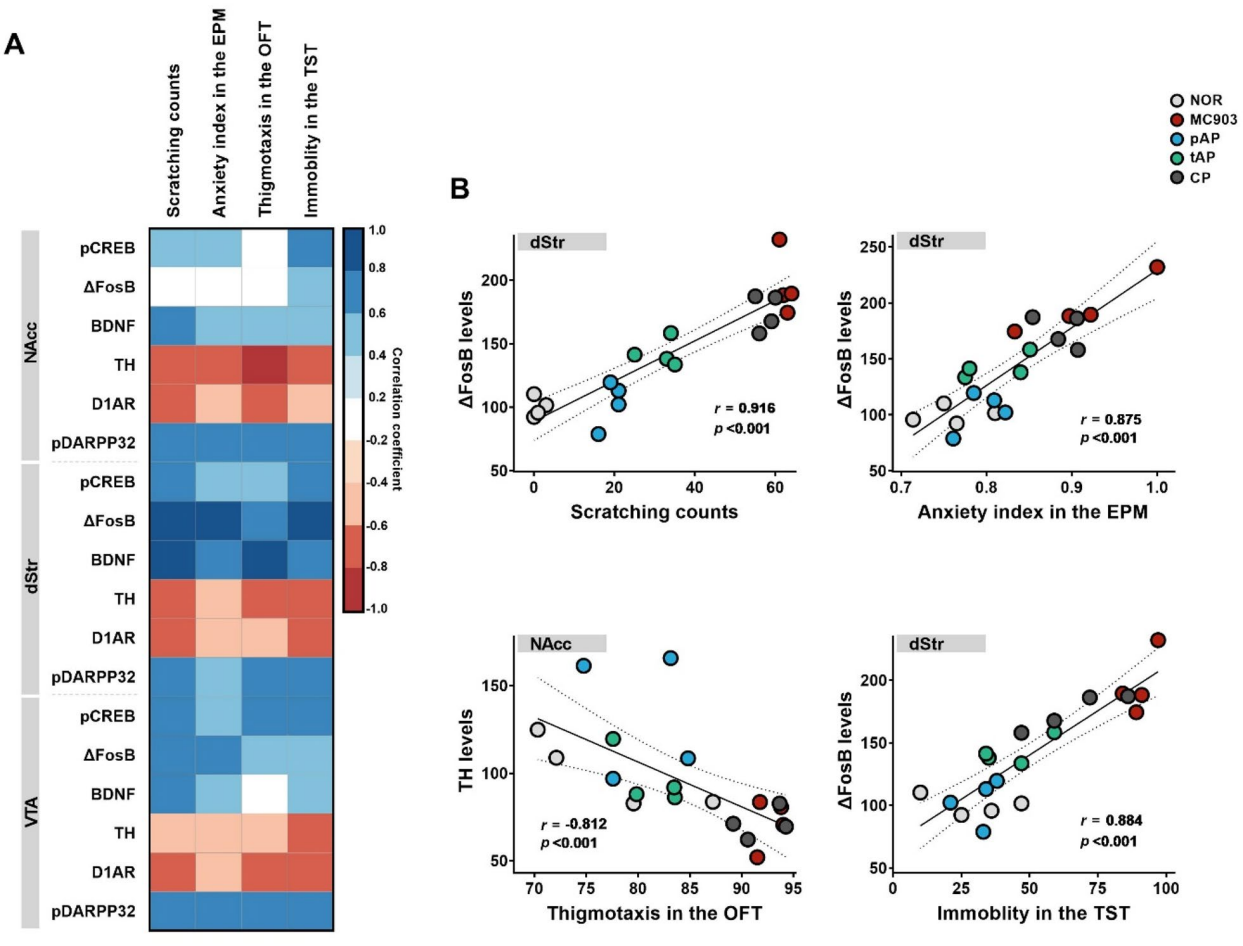
Correlations between itch- and anxiety/depression-related behavior and the expression of reward-related signaling molecules in the brain reward regions and in mice. **A** Correlation analysis heatmap comparing the itch- and anxiety/depression-related behavior to target protein expression the NAcc, dStr, and VTA. Color represents Pearson or Spearman correlation coefficients. Blue denotes a positive correlation and red a negative correlation. Non-significant correlations are left blank. **B** Scatterplots depicting the associations that showed the strong correlations for each behavioral test. For each relationship, the correlation score (*r*) and the *p* value are indicated. The linear model describing the relationship is depicted as a solid line with a 95% confidence interval given as dashed lines. *EPM* elevated plus maze, *OFT* open-field test, *TST* tail suspension test. *NAcc* nucleus accumbens, *dStr* dorsal striatum, *VTA* ventral tegmental area. *NOR* untreated control group, *MC903* MC903-induced atopic dermatitis group, *pAP* MC903- and preventive acupuncture-treated group, *tAP* MC903- and therapeutic acupuncture-treated group, *CP* MC903- and acupuncture at control point (non-acupoint)-treated group

To better understand the extent to which plasticity- and DA-related molecules in different brain reward regions were associated with itch and/or AD-related psychiatric distress, we examined the patterns of correlations for all three brain regions and for all six signaling molecules across the four behavioral tests (Fig. 5). Among the parameters of the behavioral tests for anxiety-like behavior, we used the anxiety index in the EPM and thigmotaxis in the OFT, which integrates the behavioral measurements of each test such that higher scores indicate higher anxiety-like behaviors. Higher scratching counts and immobility (time) in the TST were interpreted without any modification because it directly indicated higher itch intensity and depression-like symptoms. In all of the NAcc, dStr, and VTA, the majority of plasticity- and DA-related signaling molecules measured were statistically significantly correlated with each of the four behavioral tests; pCREB, ΔFosB, BDNF, and pDARPP32 showed positive correlations, and TH and D1AR were negatively correlated (Fig. 5A). The highest correlations for each behavioral test were of r = 0.916 for ΔFosB in dStr and scratching, 0.875 for ΔFosB in dStr and EPM, -0.812 for TH in NAcc and OFT, and 0.884 for ΔFosB in dStr and TST (Fig. 5B). Interestingly, of the six plasticity- and DA-related signaling proteins, the strongest neural adaptation-behavior correlations (for three out of four behaviors) were for striatal ΔFosB, which may reflect the superiority of striatal ΔFosB as a feature associated with the amelioration of scratching behavior as well as anxiety- and depression-like behavior in AD mice administered acupuncture.

## Discussion

The purpose of this study was to explore the impact of acupuncture on anxiety- and depression-like behaviors comorbid with AD and the biological processes that contribute to its potential impact. Our results demonstrate for the first time that acupuncture attenuates comorbid anxiety- and depression-like behaviors, aside from cutaneous inflammation and itch, and that these effects are associated with the normalization of alterations in neuronal plasticity in brain reward circuits.

First, we confirmed that treatment with MC903 led to the development of AD-like skin lesions and itching and promoted depression and anxiety-like behaviors with concomitant neuronal adaptations in brain reward circuits, as previously reported [20]. Further, acupuncture treatment at LI11 significantly alleviated AD-like skin lesions with epidermal hyperplasia and chronic itch in mice, consistent with our previous findings [25]. Moreover, we showed that the effects of acupuncture treatment go beyond improvement in AD symptoms and have meaningful impacts on comorbid anxiety- and depression-like symptoms, as indicated by the greater distance and time spent in open areas (the center of the open field and open arms in the EPM) and reduced immobility in the TST, respectively. Significant reductions in these psychiatric comorbidities were noted not only in mice administered acupuncture throughout the ongoing and post-treatment period (preventive effect) but also in those treated during the post-treatment period (therapeutic effect). Interestingly, a greater reduction in skin lesion severity and scratching behavior following acupuncture is associated with a greater reduction of concomitant anxiety- and depression-like symptoms, which supports the suggestion that psychological comorbidities may be reduced as AD improves [31].

Emerging evidence indicates that people with AD suffer from psychological stress, which can markedly increase the prevalence of anxiety and depression and in turn aggravate AD symptoms [3, 4, 32]. AD mice with anxiety- and depression-like symptoms were also in a heightened state of stress, as indicated by elevated basal corticosterone levels. Acupuncture treatment significantly reduced the increase in serum corticosterone levels following MC903 treatment, suggesting that acupuncture can be effective in treating AD animals with depression and anxiety-like phenotypes, possibly due to a reduced stress response.

Recently, it has been suggested that impairment in the brain reward system is linked to scratching-evoked pleasure, which in turn affects mental health [15, 19]. Similar to drug addiction, the pleasure of scratching, especially in chronic itch conditions, results in overactivation and ultimately, desensitization of the brain reward circuit [15, 18, 19]. This hypofunctional reward circuit may therefore elicit an amplified drive to scratch, even described as addictive [33], which is attributed to neural adaptation, requiring molecular changes in transcription and neurotrophic factors [34, 35]. In this study, repeated treatment with MC903 promoted several changes in the plasticity- and DA-related proteins in the NAcc, dStr, and VTA. First, the development of AD following MC903 treatment reduced the protein levels of the dopamine biosynthetic enzyme TH. This observation suggests that AD may reduce dopamine tone, consistent with the decreased dopamine levels in the striatum of AD mice [36]. These decreases in dopamine signaling by repeated MC903 treatment are speculated to reduce reward sensitivity, eventually leading to excessive and habitual scratching (almost to the point of compulsive behavior). Acupuncture reversed such AD-associated decreases in TH levels in these brain regions, especially in the VTA. Interestingly, stress-induced depression has been associated with reduced dopamin biosynthesis in the VTA [37]. In view of the role of the VTA in behavioral stress responses, increased dopamine biosynthesis in the VTA of AD mice following acupuncture treatment may play a role in its antidepressant effects. In contrast to TH and dopamine D1A receptor protein levels, phosphorylation levels of pDARPP-32 at Thr34 in the NAcc and dStr were significantly increased by MC903 treatment and reversed by acupuncture treatment. However, these findings are inconsistent with previous reports that the D1 receptor leads to an increased phosphorylation of DARPP32 at Thr34 in response to PKA activation and, as a result, inhibits protein phosphatase 1 (PP1), resulting in potentiation of dopaminergic signaling [38]. This discrepancy can probably be explained by the regulation of DARPP-32 phosphorylation by the signaling of other neurotransmitters such as serotonin [38]. This suggests that acupuncture may affect the signals of other neurotransmitters, as well as DA. Indeed, considerable evidence indicates that several brain neurotransmitter systems, such as serotonin, have been implicated in the modulation of the dopamine system by acupuncture [39–41].

In addition, our data showed that AD induced plasticity-related changes in the reward circuits that are associated with a depression-like phenotype, as reflected by elevations in pCREB, ΔFosB, and BDNF protein levels. These changes were markedly reversed by acupuncture treatment in MC903-induced AD mice, implying the mechanism behind the effectiveness of acupuncture in ameliorating comorbid anxiety- and depression-like behaviors in AD. ΔFosB and cAMP response element binding protein (CREB) are important transcription factors induced in the brain reward circuits after chronic exposure to pleasurable stimuli (i.e., drugs of abuse and food) [42, 43]. ΔFosB, an unusually stable, truncated splice isoform of FosB, accumulates in the brain reward circuits in response to many types of compulsive behaviors such as chronic cocaine administration, and induces cyclin-dependent kinase 5 (Cdk5) expression in these brain regions [44]. Induction of Cdk5 appears to attenuate D1 dopamine receptor signaling, at least in part, via increased phosphorylation of DARPP-32 at Thr 75, contributing to adaptive changes [45]. Thus, in our study, acupuncture may prevent an increase in ΔFosB expression in these brain regions, which in turn results in a reversal of dysregulation of dopamine signaling due to decreased D1 receptors. CREB, which is activated by phosphorylation, also underlies long-term neural adaptations associated with reward hypofunction. Its activation in the brain reward system has been linked to the development of a variety of emotional disturbances, such as depression or anxiety. In fact, elevations of CREB activity within the NAcc, via viral-mediated gene transfer or in inducible transgenic mice, produce anhedonia-like signs. On the other hand, reductions in CREB activity in the NAcc, through viral-or transgenic-mediated expression of a dominant negative CREB mutant or through knockdown of CREB in the NAcc of floxed CREB mice causes opposite changes [46]. BDNF, a downstream target of CREB, also plays an important role in behavioral plasticity and modulation of reward through its signaling actions in the brain reward system [47]. Increased BDNF signaling in the VTA-NAc mesolimbic pathway promotes a depression-like phenotype, whereas viral-mediated local knockdown of BDNF in the VTA has the opposite effect [48]. With this in mind, we speculate that acupuncture may modulate the enhanced signaling of both pCREB and BDNF levels in the brain reward circuit of MC903-induced AD mice with comorbid anxiety and depression-like behaviors. Consistent with this possibility, acupuncture treatment decreased AD-evoked increases in both pCREB and BDNF levels in these regions. These findings may explain how acupuncture reduces psychological comorbidities in AD.

To better understand which plasticity- and DA-related protein in different brain regions is associated with behavior, we examined the patterns of correlation for all different brain reward regions (the NAcc, dStr and VTA), for all six signaling molecules, across the four behavioral tests. Interestingly, of the six neuroplasticity- and DA-related proteins, dorsal striatal ΔFosB showed the strongest correlation with each behavior (3 out of 4 behaviors), which may reflect the role of ΔFosB in the dStr as a risk for emotional impairment in AD and in the mechanism of acupuncture treatment for comorbid anxiety- and depressive-like behaviors of AD.

## Conclusion

In summary, our results demonstrated that acupuncture had a significant positive impact on comorbid anxiety- and depression-like behaviors by modulating neuroadaptation in the brain reward circuit in mice with AD. Although the causative mechanisms underlying the beneficial effects of acupuncture on negative emotions in AD-associated comorbidities remain to be determined, these findings suggest that acupuncture could be considered for the non-pharmacological management of people with AD experiencing anxiety or depression symptoms.

## Materials and methods

### Animals

Male C57BL/6 mice (7 weeks old) from Daehan BioLink (Korea) were housed in cages of 4–5 under standard housing conditions of a 12-h light: dark cycle with free access to food and water. All procedures were conducted in accordance with the Kyung Hee University Guidelines for the Care and Use of Laboratory Animals and approved by the Kyung Hee University Animal Care Committee for Animal Welfare (IACUC-2017-024-1).

### Induction of AD-like skin lesions using MC903

**Fig. 6:**
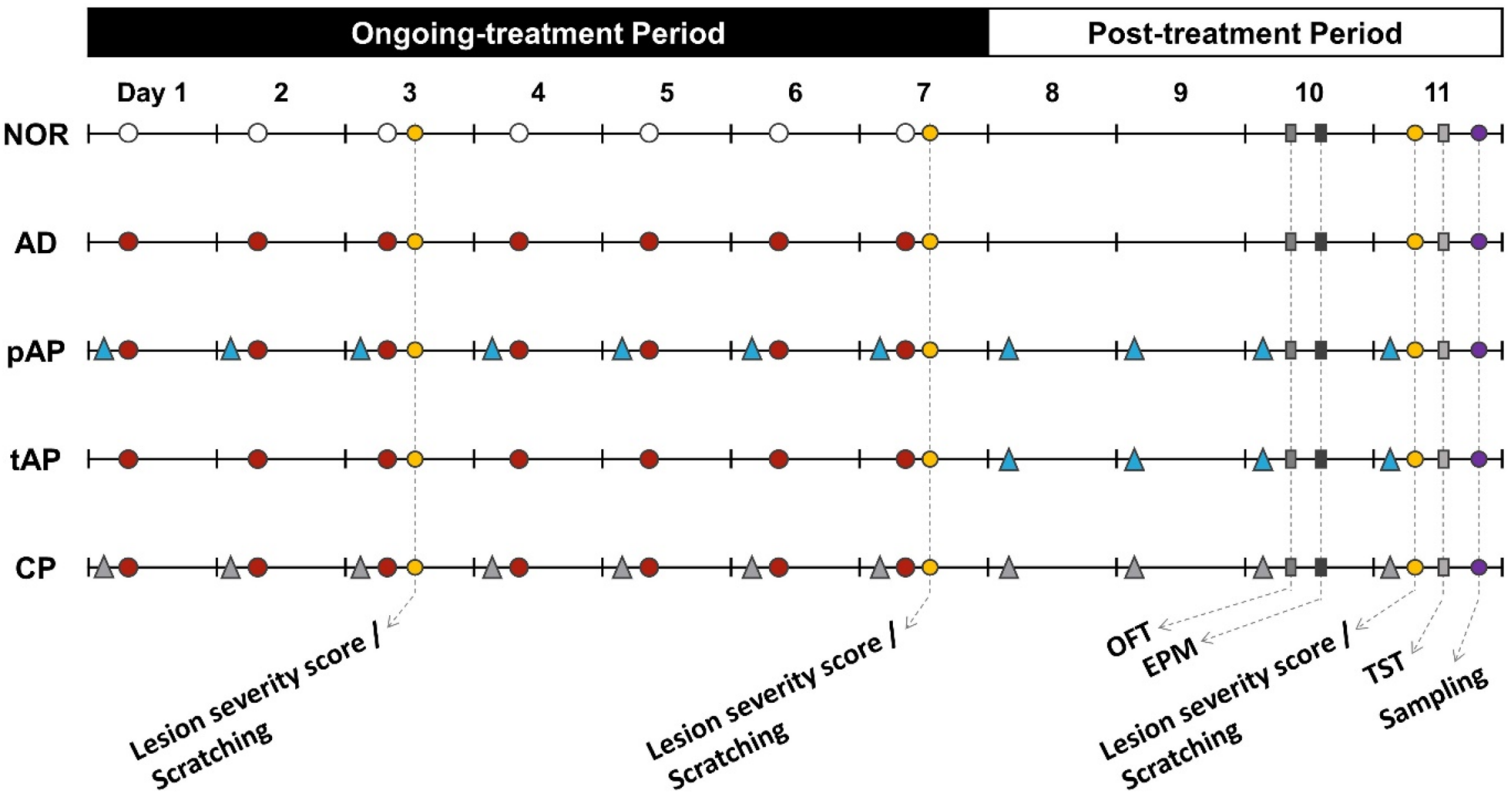
Schematic overview for overall experimental design applied to each group of animals. *NOR* untreated control group, *MC903* MC903-induced atopic dermatitis group, *pAP* MC903- and preventive acupuncture-treated group, *tAP* MC903- and therapeutic acupuncture-treated group, *CP* MC903- and acupuncture at control point (non-acupoint)-treated group

The right cheek was shaved two days before starting the experiments under brief anesthesia with isoflurane. To induce AD-like lesions, a vitamin D analog, MC903 (1.65 µg in 20 µl ethanol; Sigma-Aldrich, USA), was applied topically on the right cheek once a day for 7 days. Control mice were treated similarly with vehicle (ethanol). Lesion severity, scratching behavior, and anxiety- and depression-like behaviors were observed on days 10 and 11 after the first injection of MC903. A detailed schedule is shown in Fig. 6.

### Acupuncture treatment

Gok-Ji (LI11) is a common acupoint used to treat eczema [49]. We have previously published results on the effectiveness of acupuncture at the LI11 acupoint for reducing AD-related inflammation and itch [25]. Thus, we chose LI11 in this study to determine the impact of acupuncture on comorbid anxiety- and depression-like behaviors in AD. Acupuncture was performed at the bilateral LI11 acupoints located in the depression on the lateral end of the cubital crease when the elbow was fully flexed [25]. Mice were held gently by hand, and a small disposable sterile acupuncture needle (0.18 × 8 mm; Haenglim-Seoweon Acuneedle Co., Korea) was inserted at a depth of 2–3 mm into the LI11. The needle was twisted for 30 s at a rate of twice per second and immediately removed. The same procedure was applied to control points (non-acupoints) located on the gluteus muscle, 1 cm distal to the tail base [25]. Each animal received acupuncture once a day immediately before treatment with MC903, either during the ongoing treatment and post-treatment period (pAP group) or during the post-treatment period (tAP group). Mice in the CP group received acupuncture at control points once a day just before treatment with MC903 during the ongoing treatment and post-treatment periods. Mice in the NOR and MC903 groups were also slightly grabbed for 60 s without acupuncture. A detailed schedule is shown in Fig. 6.

### Skin lesion severity

The severity of AD-like skin lesions was scored as described previously [25]. Briefly, the skin lesion severity scores were assessed for redness (erythema), dryness (xerosis), and scabbing (excoriation) based on a 0–3 scale (0 = none; 1 = mild; 2 = moderate; 3 = severe) and summed.

### Quantification of scratching behavior

Scratching behavior was quantified by recording the behavior and scoring videos manually. Mice were first habituated to transparent plastic cylinders (11 cm in diameter; one mouse/cylinder) for 30 min prior to the beginning of recording. Immediately after acupuncture treatment, the mice were returned to the same cylinder and scratching was recorded for 30 min. The time spent scratching and the total number of scratch bouts were quantified over a 30 min period. One bout of scratching was defined as an episode in which a mouse lifted its hind paw and scratched continuously for any length of time until the paw returned to the floor. Behavior was counted by a blinded scorer.

### Histological evaluation

Formalin-fixed skin samples were embedded in paraffin, cut into 5 μm-thick sections, and stained with hematoxylin-eosin for histological examination of the skin. The extent of epidermal hyperplasia was assessed as the epidermal thickness of the lesional skin measured using ImageJ software.

### Open field test

Mice were placed in the center of a white plastic box (40 × 40 × 27 cm) in a dimly lit room and allowed to explore for 5 min. Movement was tracked using SMART v3.0 (Panlab, Barcelona, Spain). The box was cleaned with 70% ethanol and water between tests. Measurements included the time spent and the distance traveled in the central zone. Thigmotaxis, the tendency to remain close to the walls, has been validated as a measure of anxiogenic behavior in mice; thigmotaxis increases as anxiety levels increase. It was assessed by the percentage of distance moved in the outer zone to the total distance moved.

### Elevated plus maze

A plus maze apparatus consisting of two open arms (16 × 5 cm) and two enclosed arms (16 × 5 cm with 12 cm high walls) that extended from a central platform (5 × 5 cm) was placed 40 cm above the floor. Each mouse was placed in the center of the elevated plus maze (EPM) facing an open arm away from the experimenter and allowed to freely explore the maze for 5 min in dim lighting. Movement was tracked using SMART v3.0 (Panlab, Barcelona, Spain). The box was cleaned with 70% ethanol and water between tests. The number of entries and time spent in the closed and open arms were monitored for 10 min and analyzed using SMART v3.0 (Panlab, Barcelona, Spain). The percentage of open arm entries and percentage of open arm time were used as indices of anxiety-like behavior. The percentage of open arm entries was calculated as the number of open arm entries divided by total entries (open arm entries plus closed arm entries). The open arm time percentage was calculated as the amount of time spent in open arms divided by the total amount of time spent in both open and closed arms. An anxiety index ranging from 0 (low anxiety) to 1 (high anxiety) was also calculated based on the following formula [20]: anxiety index = 1 − [(time spent in open arm/test duration + number of entries to the open arms/total number of entries)/2].

### Tail suspension test

The tail suspension test (TST) was performed as described previously [20]. Mice were suspended by their tail with adhesive tape from a horizontal bar placed approximately 50 cm above the surface, and video was recorded for 6 min. The amount of time each mouse spent immobile was scored during the entire 6 min test.

### Serum corticosterone levels

Blood samples were collected through cardiac puncture, followed by cervical dislocation to ensure death. Serum was obtained after allowing the blood to clot for 30 min at room temperature, followed by centrifugation at 1000 × g for 15 min. Serum corticosterone levels were measured using a commercially available corticosterone ELISA kit (Abcam, Cambridge, MA, USA), according to the manufacturer’s instructions.

### Immunoblot assay

The levels of CREB, pCREB, ΔFosB, BDNF, TH, D1AR, DARPP-32, pDARPP-32, and β-actin were analyzed by immunoblotting. Briefly, mouse brains were quickly removed immediately after blood collection and sliced into 1-mm coronal sections using a mouse brain matrix. The NAcc, dStr, and VTA were microdissected bilaterally by using brain tissue punches (Stoelting, USA). Brain tissue lysates from the NAcc, dStr, and VTA were prepared in a cocktail of lysis buffer (CyQUANT; Invitrogen, USA), protease inhibitors (Roche Applied Science, Germany) and PhosStop phosphatase inhibitors (Roche Applied Science). Protein concentrations were determined using the BCA Protein Assay Kit (Thermo Scientific, USA). Equal amounts of total protein were resolved on 10% polyacrylamide gel electrophoresis (PAGE) gels and transferred to PVDF membranes. Following incubation with antibodies against pCREB (1:500; Cell Signaling Technology, USA), total CREB (1:500; Cell Signaling Technology), ΔFosB (1:500; Cell Signaling Technology), BDNF (1:300; Santa Cruz Biotechnology, USA), TH (1:1000; Santa Cruz Biotechnology), D1AR receptor (1:250; Santa Cruz Biotechnology), pDARPP-32 (1:100; Cell Signaling Technology), total DARPP-32 (1:100; Cell Signaling Technology), or β-actin (Sigma-Aldrich), blots were visualized using the SuperSignal West Pico Chemiluminescent Substrate (Thermo Scientific). Band intensities of scanned films were quantified by densitometry using ImageJ software. Phosphoproteins were normalized to their respective total proteins, and non-phosphoproteins were normalized to the β-actin loading control. Changes in protein levels are presented as percentages relative to the controls, which were denoted as 100%.

### Statistical analysis

All data are presented as mean ± SD, with individual values indicated in each figure. All statistical analyses were performed using GraphPad Prism 9.3.1 (GraphPad Software, San Diego, CA, USA). All data were tested for normal distribution using the Shapiro-Wilk test and homogeneity of variances using Bartlett’s test. When assumptions of normal distribution and homogeneity of variances were met, ordinary one-way ANOVA with Tukey’s *post-hoc* test was used for multiple comparisons. The Kruskal-Wallis test with Dunn’s multiple comparisons or Welch-ANOVA with Dunnett’s T3 multiple comparisons test was used for multiple comparisons involving data that did not pass the Shapiro-Wilk test. Time-course data were compared using repeated two-way ANOVA with Tukey’s multiple comparisons. Associations between behavioral phenotypes and plasticity- and DA-related molecules were analyzed using Pearson or Spearman correlation tests depending on the normal distribution of the residuals. Differences between the groups with *p* < 0.05 were considered statistically significant.

## Supplementary Information

**Additional file 1: Fig. S1. **Representative images of hematoxylin and eosin–stained skin sections from each experimental group of mice on day 11. *NOR* untreated control group, *MC903* MC903-induced atopic dermatitis group, *pAP* MC903- and preventive acupuncture-treated group, *tAP* MC903- and therapeutic acupuncture-treated group, *CP* MC903- and acupuncture at control point (non-acupoint)-treated group. Magnification, ×100. **Fig. S2. **Representative activity tracks in the open field test (OFT, top) and elevated plus maze (EPM, bottom). *NOR* untreated control group, *MC903* MC903-induced atopic dermatitis group, *pAP* MC903- and preventive acupuncture-treated group, *tAP* MC903- and therapeutic acupuncture-treated group, *CP* MC903- and acupuncture at control point (non-acupoint)-treated group. **Fig. S3. **Representative immunoblots of plasticity-related (**A**, **C**, and **E**; pCREB, ΔFosB and BDNF) and DA-related (B, D, and F; TH, D1AR and pDARPP-32) proteins in the brain reward regions. *NOR* untreated control group, *MC903* MC903-induced atopic dermatitis group, *pAP* MC903- and preventive acupuncture-treated group, *tAP* MC903- and therapeutic acupuncture-treated group, *CP* MC903- and acupuncture at control point (non-acupoint)-treated group. *CREB* cyclic AMP-response element binding protein, *pCREB* phospho-Ser133 CREB, *BDNF* brain-derived neurotrophic factor, *TH* tyrosine hydroxylase, *D1AR* dopamine D1 receptor, * DARPP-32* dopamine- and cAMP-regulated phosphoprotein of 32 kDa, *pDARPP-32* phospho-Thr34 DARPP-32. **Fig. S4. C**orrelations between clinical symptom of atopic dermatitis and psychological distress. **A** Heatmap of the correlation matrix comparing the clinical symptoms of atopic dermatitis (skin lesion severity and scratching behavior) and psychological distress (anxiety-like/depression-like behavior). Color represents the Spearman’s correlation coefficient. *** *p* < 0.001. **B** Scatterplots depicting the association between pairs. The linear model describing the relationship is depicted as a solid line with a 95% confidence interval (CI), as indicated by dashed lines. *EPM* elevated plus maze, *OFT* open-field test, *TST* tail suspension test. *NOR* untreated control group, *MC903* MC903-induced atopic dermatitis group, *pAP* MC903- and preventive acupuncture-treated group, *tAP* MC903- and therapeutic acupuncture-treated group, *CP* MC903- and acupuncture at control point (non-acupoint)-treated group.

## Data Availability

All data generated or analysed during this study are included in this published article [and its supplementary information files].
