# Correction: Acupuncture attenuates comorbid anxiety- and depressive-like behaviors of atopic dermatitis through modulating neuroadaptation in the brain reward circuit in mice

**DOI:** 10.1186/s40659-022-00402-5

**Published:** 2022-11-26

**Authors:** Mijung Yeom, Sora Ahn, Sun-Young Jang, Jae-Hwan Jang, Youngrye Lee, Dae-Hyun Hahm, Hi-Joon Park

**Affiliations:** 1 Acupuncture and Meridian Science Research Center (AMSRC), College of Korean Medicine, Kyung Hee University, 02447 Seoul, Republic of Korea; 2 Department of Meridian Medical Science, College of Korean Medicine, Graduate School, Kyung Hee University, 02447 Seoul, Republic of Korea; 3 Department of Physiology, School of Medicine, Kyung Hee University, 02447 Seoul, Republic of Korea; 4 BioNanocomposite Research Center, Kyung Hee University, 02447 Seoul, Republic of Korea; 5 Department of Anatomy & Information Sciences, College of Korean Medicine, Kyung Hee University, 02447 Seoul, Republic of Korea

## Correction: Biological Research (2022) 55:28 https://doi.org/10.1186/s40659-022-00396-0

Following publication of the original article, the authors identified that they provided the wrong Funding Information. The correct grant reference number is 2020R1A4A1018598.

The original article [1] has been revised.
