# Supplementary material for: Acupuncture attenuates comorbid anxiety- and depressive-like behaviors of atopic dermatitis through modulating neuroadaptation in the brain reward circuit in mice
Source: Biol Res. 2022 Sep 10;55:28. doi: 10.1186/s40659-022-00396-0 (PMC9463810; doi:10.1186/s40659-022-00396-0)
Supplement: Supplementary file 1 — Additional file 1: Fig. S1. Representative images of hematoxylin and eosin–stained skin sections from each experimental group of mice on day 11. NOR untreated control group, MC903 MC903-induced atopic dermatitis group, pAP MC903- and preventive acupuncture-treated group, tAP MC903- and therapeutic acupuncture-treated group, CP MC903- and acupuncture at control point (non-acupoint)-treated group. Magnification, ×100. Fig. S2. Representative activity tracks in the open field test (OFT, top) and elevated plus maze (EPM, bottom). NOR untreated control group, MC903 MC903-induced atopic dermatitis group, pAP MC903- and preventive acupuncture-treated group, tAP MC903- and therapeutic acupuncture-treated group, CP MC903- and acupuncture at control point (non-acupoint)-treated group. Fig. S3. Representative immunoblots of plasticity-related (A, C, and E; pCREB, ΔFosB and BDNF) and DA-related (B, D, and F; TH, D1AR and pDARPP-32) proteins in the brain reward regions. NOR untreated control group, MC903 MC903-induced atopic dermatitis group, pAP MC903- and preventive acupuncture-treated group, tAP MC903- and therapeutic acupuncture-treated group, CP MC903- and acupuncture at control point (non-acupoint)-treated group. CREB cyclic AMP-response element binding protein, pCREB phospho-Ser133 CREB, BDNF brain-derived neurotrophic factor, TH tyrosine hydroxylase, D1AR dopamine D1 receptor, DARPP-32 dopamine- and cAMP-regulated phosphoprotein of 32 kDa, pDARPP-32 phospho-Thr34 DARPP-32. Fig. S4. Correlations between clinical symptom of atopic dermatitis and psychological distress. A Heatmap of the correlation matrix comparing the clinical symptoms of atopic dermatitis (skin lesion severity and scratching behavior) and psychological distress (anxiety-like/depression-like behavior). Color represents the Spearman’s correlation coefficient. *** p < 0.001. B Scatterplots depicting the association between pairs. The linear model describing the relationship is depicted as a solid line with a [file 40659_2022_396_MOESM1_ESM.docx]

**Additional file 1 Information**

**Additional file 1: Fig. S1. Representative images of hematoxylin and eosin–stained skin sections from each experimental group of mice on day 11.** NOR, untreated control group; MC903, MC903-induced atopic dermatitis group; pAP, MC903- and preventive acupuncture-treated group; tAP, MC903- and therapeutic acupuncture-treated group; CP, MC903- and acupuncture at control point (non-acupoint)-treated group. Magnification, ×100.

**Additional file 1: Fig. S2.** **Representative activity tracks in the open field test (OFT, top) and elevated plus maze (EPM, bottom).** NOR, untreated control group; MC903, MC903-induced atopic dermatitis group; pAP, MC903- and preventive acupuncture-treated group; tAP, MC903- and therapeutic acupuncture-treated group; CP, MC903- and acupuncture at control point (non-acupoint)-treated group.

**Additional file 1: Fig. S3. Representative immunoblots of plasticity-related (A, C, and E; pCREB, ΔFosB and BDNF) and DA-related (B, D, and F; TH, D1AR and pDARPP-32) proteins in the brain reward regions.** NOR, untreated control group; MC903, MC903-induced atopic dermatitis group; pAP, MC903- and preventive acupuncture-treated group; tAP, MC903- and therapeutic acupuncture-treated group; CP, MC903- and acupuncture at control point (non-acupoint)-treated group. cyclic AMP-response element binding protein, CREB; phospho-Ser133 CREB, pCREB; brain-derived neurotrophic factor, BDNF; tyrosine hydroxylase, TH; dopamine D1 receptor, D1AR; dopamine- and cAMP-regulated phosphoprotein of 32 kDa, DARPP-32, phospho-Thr34 DARPP-32, pDARPP-32.

**Additional file 1: Fig. S4. Correlations between clinical symptom of atopic dermatitis and psychological distress**. (A) Heatmap of the correlation matrix comparing the clinical symptoms of atopic dermatitis (skin lesion severity and scratching behavior) and psychological distress (anxiety-like/depression-like behavior). Color represents the Spearman’s correlation coefficient. *** *p* < 0.001. (B) Scatterplots depicting the association between pairs. The linear model describing the relationship is depicted as a solid line with a 95% confidence interval (CI), as indicated by dashed lines. EPM, elevated plus maze; OFT, open-field test; TST, tail suspension test. NOR, untreated control group; MC903, MC903-induced atopic dermatitis group; pAP, MC903- and preventive acupuncture-treated group; tAP, MC903- and therapeutic acupuncture-treated group; CP, MC903- and acupuncture at control point (non-acupoint)-treated group.

**Supplemental Figure S1.**

**
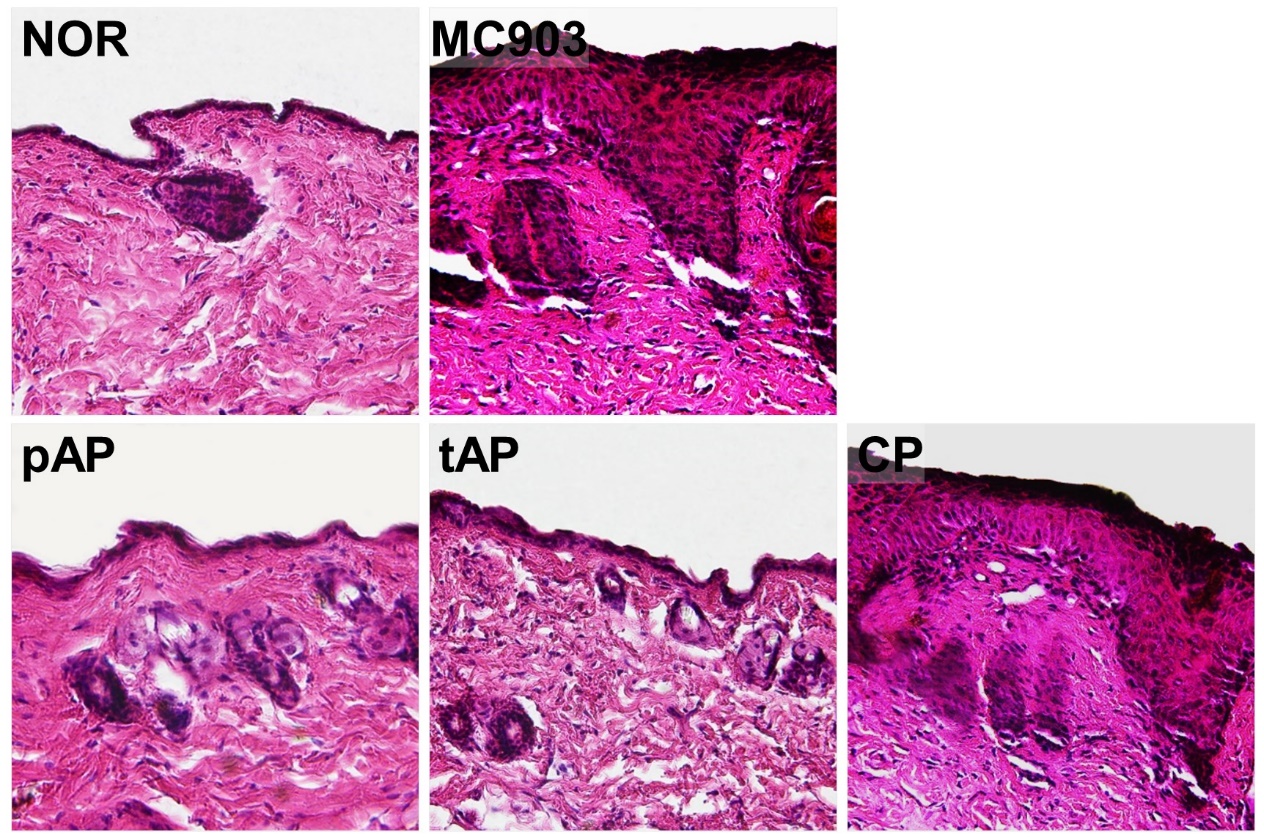
**

**Supplemental Figure S2.**

**
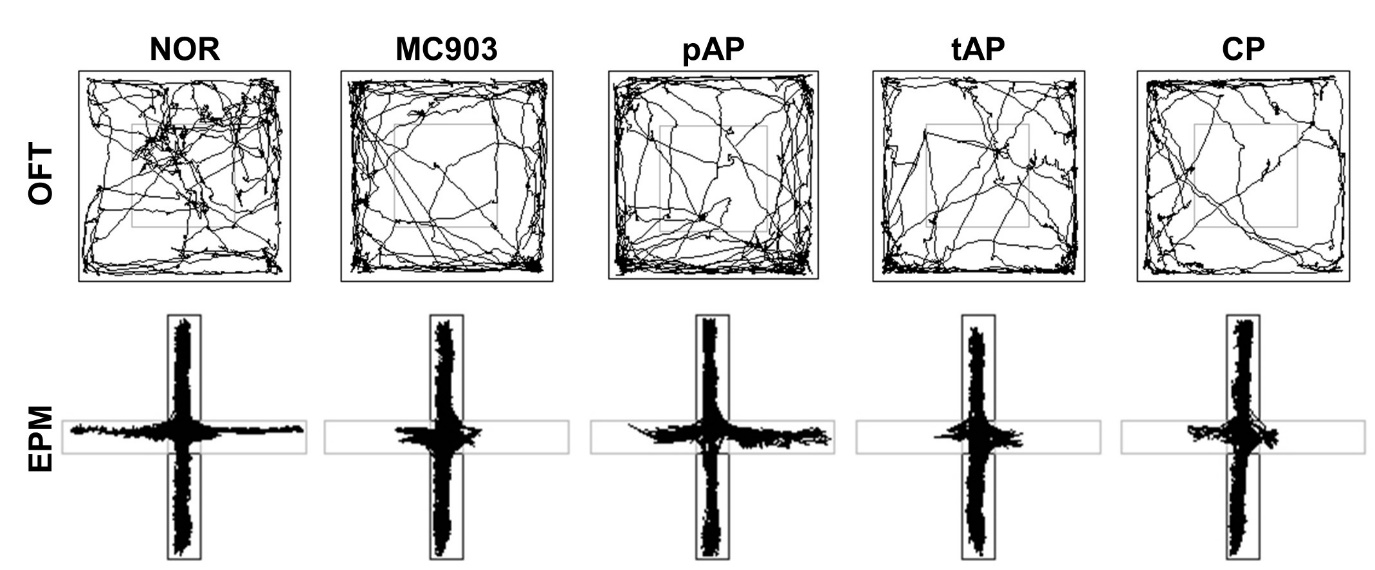
**

**Supplemental Figure S3.**

**
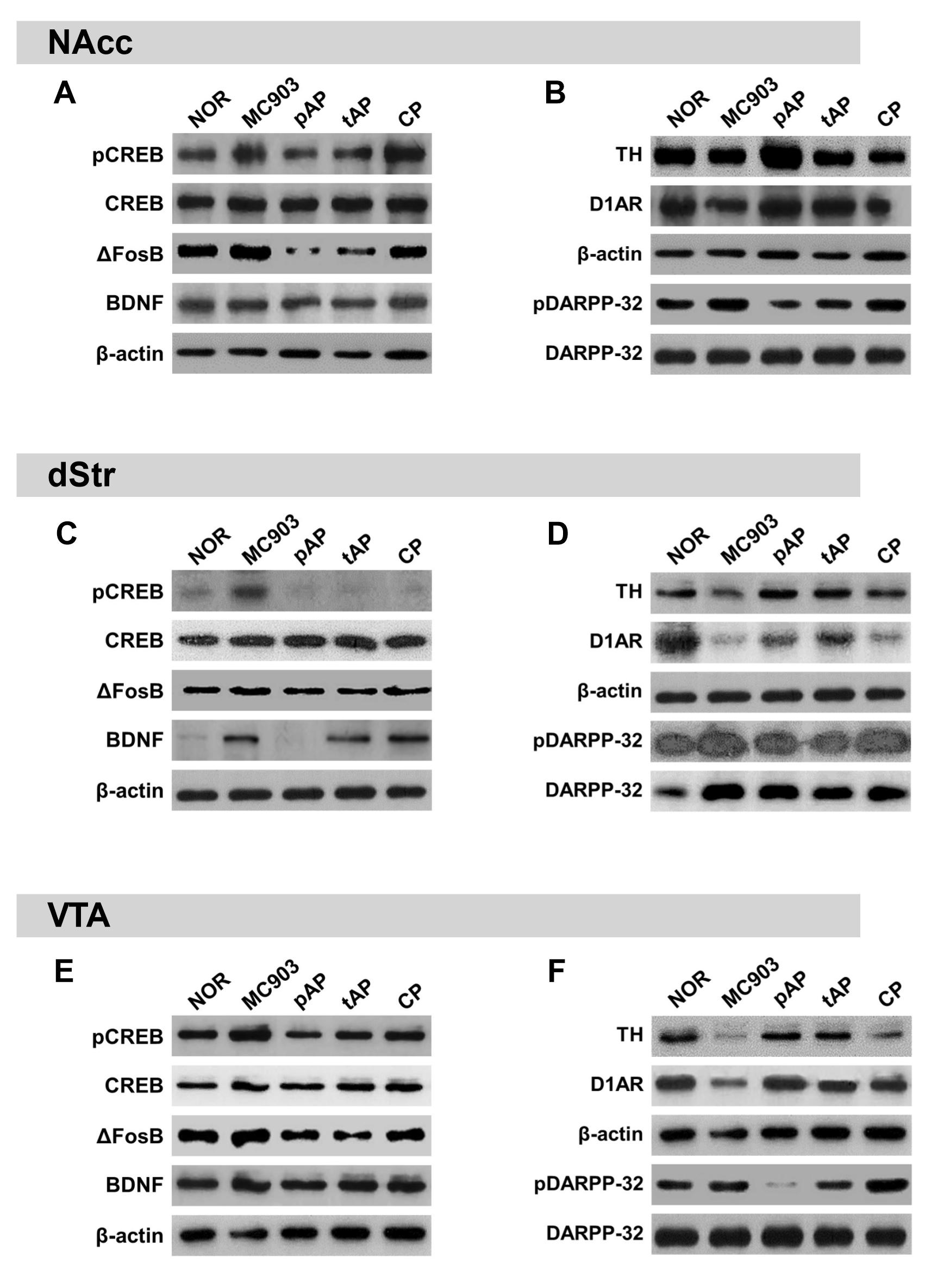
**

**Supplemental Figure S4.**

**
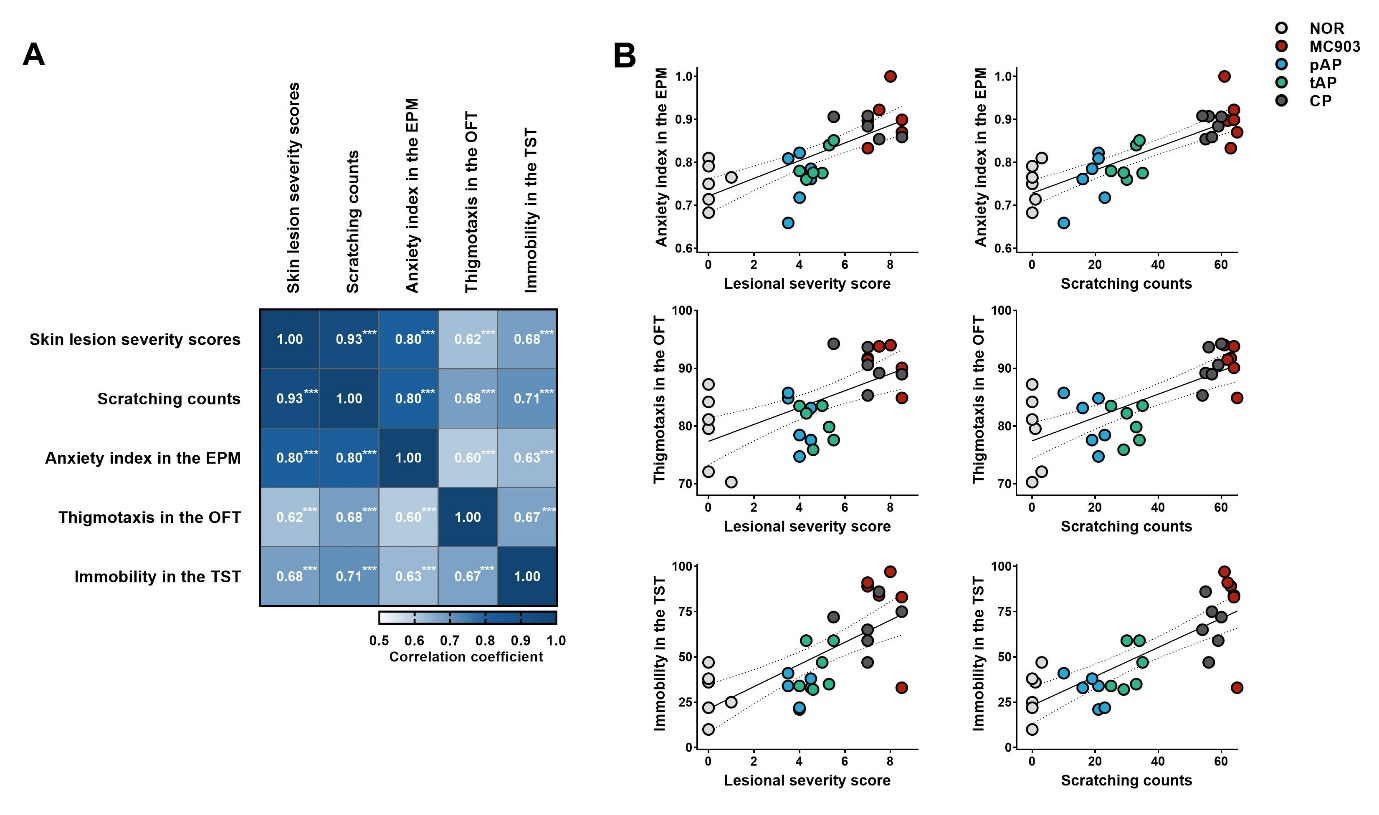
**
